# Elucidating the CXCL12/CXCR4 Signaling Network in Chronic Lymphocytic Leukemia through Phosphoproteomics Analysis

**DOI:** 10.1371/journal.pone.0011716

**Published:** 2010-07-22

**Authors:** Morgan O'Hayre, Catherina L. Salanga, Thomas J. Kipps, Davorka Messmer, Pieter C. Dorrestein, Tracy M. Handel

**Affiliations:** 1 Skaggs School of Pharmacy and Pharmaceutical Sciences, University of California San Diego, La Jolla, California, United States of America; 2 Rebecca and John Moores Cancer Center, University of California San Diego, La Jolla, California, United States of America; Health Canada, Canada

## Abstract

**Background:**

Chronic Lymphocytic Leukemia (CLL) pathogenesis has been linked to the prolonged survival and/or apoptotic resistance of leukemic B cells *in vivo*, and is thought to be due to enhanced survival signaling responses to environmental factors that protect CLL cells from spontaneous and chemotherapy-induced death. Although normally associated with cell migration, the chemokine, CXCL12, is one of the factors known to support the survival of CLL cells. Thus, the signaling pathways activated by CXCL12 and its receptor, CXCR4, were investigated as components of these pathways and may represent targets that if inhibited, could render resistant CLL cells more susceptible to chemotherapy.

**Methodology/Principal Findings:**

To determine the downstream signaling targets that contribute to the survival effects of CXCL12 in CLL, we took a phosphoproteomics approach to identify and compare phosphopeptides in unstimulated and CXCL12-stimulated primary CLL cells. While some of the survival pathways activated by CXCL12 in CLL are known, including Akt and ERK1/2, this approach enabled the identification of additional signaling targets and novel phosphoproteins that could have implications in CLL disease and therapy. In addition to the phosphoproteomics results, we provide evidence from western blot validation that the tumor suppressor, programmed cell death factor 4 (PDCD4), is a previously unidentified phosphorylation target of CXCL12 signaling in all CLL cells probed. Additionally, heat shock protein 27 (HSP27), which mediates anti-apoptotic signaling and has previously been linked to chemotherapeutic resistance, was detected in a subset (∼25%) of CLL patients cells examined.

**Conclusions/Significance:**

Since PDCD4 and HSP27 have previously been associated with cancer and regulation of cell growth and apoptosis, these proteins may have novel implications in CLL cell survival and represent potential therapeutic targets. PDCD4 also represents a previously unknown signaling target of chemokine receptors; therefore, these observations increase our understanding of alternative pathways to migration that may be activated or inhibited by chemokines in the context of cancer cell survival.

## Introduction 

B cell Chronic Lymphocytic Leukemia (CLL) is an adult leukemia characterized by the accumulation of B cells in the blood, bone marrow and secondary lymphoid tissues due to apparent survival advantages and/or apoptosis resistance of these cells *in vivo* [1]. There is significant heterogeneity in the disease progression between CLL patients. A more aggressive form of the disease, which results in lower patient survival time, correlates with markers including unmutated immunoglobulin heavy chain variable region (IgHV) status and high expression of the tyrosine kinase ZAP-70 (ZAP-70+). Although the accumulation of a monoclonal population of CD5+/CD19+ B cells is characteristic of both prognostic groups, aggressive CLL appears to have some distinct characteristics and signaling properties compared to indolent CLL [2].

**Figure 1:**
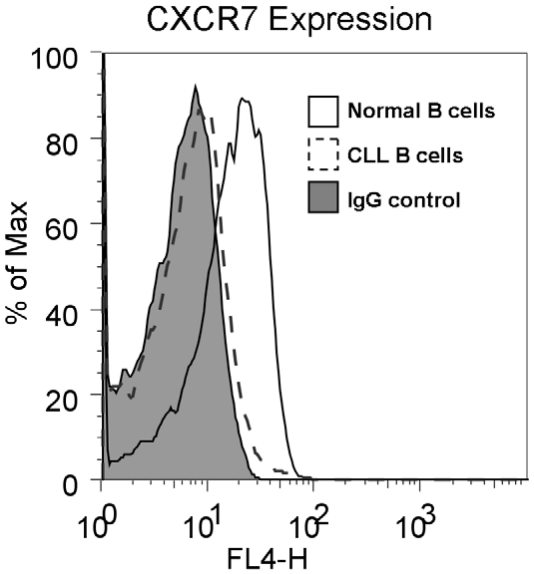
CXCR7 Expression on Normal B cells and CLL B Cells. Surface CXCR7 expression on Normal B cells (solid line) and CLL B cells (dashed line) was analyzed by flow cytometry and referenced to an IgG1 isotype control (filled histogram). Profiles are representative of B cells from 4 healthy donors (normal B cells) and 10 CLL patients' B cells.

Despite their enhanced survival *in vivo*, when CLL cells from patients are cultured *in vitro*, they rapidly undergo apoptosis under conditions that support the survival of normal B cells, underscoring the dependence of these cells on survival cues from the microenvironment [3], [4]. In the microenvironment, marrow stromal cells are believed to secrete factors that promote CLL cell survival in patients; correspondingly, when monocytes isolated from peripheral blood of CLL patients are cultured, they differentiate into “Nurse-like cells” (NLCs) that promote CLL survival *in vitro* [3]. One of the factors known to be secreted by these NLCs and to support CLL survival, is the chemokine, CXCL12 (SDF-1). Additionally, CXCR4, the receptor for CXCL12, is overexpressed on CLL cells compared to normal B cells, and thus has the potential for enhanced responsiveness to CXCL12 signaling [5]. Although another chemokine receptor, CXCR7, can also bind CXCL12 and was previously shown to be expressed on B cells [6], surface expression of CXCR7 was not observed on CLL B cells (Figure 1). Therefore the CXCL12 signaling effects are likely mediated exclusively by CXCR4 in these cells.

While chemokines and their G-protein coupled receptors are best known for their role in directing the migration of immune cells, it is clear that these proteins are involved in many other biological functions. The CXCL12/CXCR4 axis is critical for developmental processes including lymphopoiesis, and central nervous system and cardiac development, and knockout of either the ligand or receptor in mice results in embryonic lethality [7]. Due to the involvement of CXCL12/CXCR4 in migration, angiogenesis, and development, it is not surprising that this axis is often exploited by cancer cells for metastasis as well as survival and proliferation [8]. However, the specific molecular mechanisms by which these various functions are effectuated and how these signaling pathways target different downstream signaling molecules in cancer cells compared to non-malignant counterpart cells is largely unknown. Similarly, while it is known that Akt and ERK1/2 are activated by CXCL12 in CLL, the downstream targets of these pathways and activation of other pathways have not been elucidated [9].

**Figure 2:**
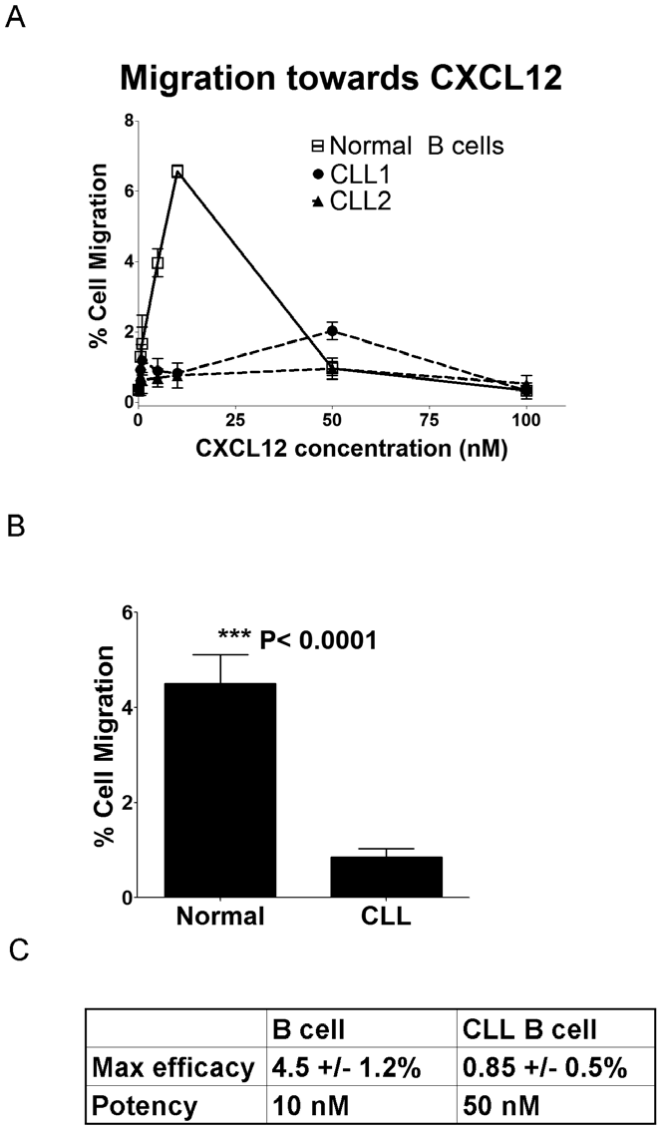
CXCL12-mediated Migration of CLL B Cells and Normal B Cells. A) Representative migration assay profiles for normal B cells (solid line) compared to 2 different CLL patients' cells (dashed lines) performed in triplicate over a range of 0–100 nM CXCL12 and normalized to no chemokine. Data represents the percent of cells migrated through the transwell filter. B) Bar graph comparing the maximum percent cell migration observed in normal B cells and CLL B cells. Data represents an average of multiple transwell migration assays from separate donors, normal B cells *n* = 5, CLL B cells *n* = 7, each performed in triplicate. Differences were found to be statistically significant (p<0.0001) based on Student's t-test. C) Table depicting the maximum percent migration to CXCL12 (efficacy) and the concentration at which maximal migration is achieved (potency) observed in normal B cells compared to CLL B cells.

Despite the upregulation of CXCR4 and strong Akt and ERK signaling demonstrated by CLL cells in response to CXCL12, the CLL cells actually migrate less efficiently to CXCL12 than B cells from healthy donors in a transwell migration assay (Figure 2). Thus, in CLL cells, it appears that signaling downstream of CXCL12/CXCR4 may be redirected towards survival signaling in lieu of cell migration. To better characterize the signaling responses to CXCL12 stimulation, primary CLL cells isolated from 5 patients were subjected to phosphoproteomic analysis by liquid chromatography and tandem mass spectrometry (LC-MS/MS). Rather than attempting to characterize the complete phosphoproteome of CLL cells, this approach was designed to generate new hypotheses about the CXCL12/CXCR4 signaling network in CLL survival, and to identify downstream proteins that might be good therapeutic targets. While many phosphoproteins were identified in the CLL cells, comparison of spectral counts between CXCL12 stimulated and unstimulated cells allowed identification of proteins phosphorylated as a consequence of CXCL12/CXCR4 signaling. With follow-up experiments, the tumor suppressor PDCD4 was validated as a downstream phosphorylation target of CXCL12 signaling in all CLL patient cells examined (n = 10) and HSP27 was similarly validated in a subset of CLL patients (∼25%). Although these proteins have been previously linked to cancer cell survival, they have not been previously associated with CLL nor has PDCD4 been established as a downstream phosphorylation target of CXCL12 signaling. Furthermore, a number of other proteins (many of which do not have commercially available phospho-specific antibodies available) have been proposed as potential downstream phosphorylation targets of CXCL12 stimulation based on spectral count analysis of the CLL phosphoproteomics data.

## Methods

### Cells and reagents

Peripheral blood mononuclear cells (PBMCs) were obtained from leukopheresis samples of CLL patients following written consent at the Rebecca and John Moores Cancer Center at the University of California San Diego (UCSD), in compliance with the Declaration of Helsinki. These studies were approved by the Institutional Review Board of UCSD. PBMCs were isolated by Ficoll-Paque (GE Healthcare) density gradient centrifugation as previously described [9]. The isolated PBMCs were used fresh and cultured for phosphoproteomics analysis or frozen as liquid nitrogen stocks in 90% heat inactivated fetal bovine serum (FBS)/10% DMSO for follow-up analysis by western blot. PBMCs used in the proteomics experiments were determined to be >90% CLL B cells as assessed by CD5+/CD19+ staining and flow cytometry analysis. For western blot validation, CLL B cells were purified from the PBMCs by negative selection using the magnetic associated cell sorting (MACS) (Miltenyi Biotec, Auburn, CA) by depletion of CD14+ (monocytes) and CD2+ (T cells) cells, leading to >99% CLL B cell purity. Normal B cells were purified from PBMCs from healthy donors (San Diego Blood Bank) using the MACS B cell Isolation Kit II (Miltenyi Biotec, Auburn, CA) according to the manufacturer's protocol and were determined to be >90% pure by flow analysis staining for CD19+/CD3-/CD14- cells. RPMI-1640 glutamax media and FBS were obtained from Gibco (Invitrogen, Carlsbad, CA).

### Recombinant CXCL12 preparation

CXCL12 was expressed recombinantly in BL21 *E. coli* as previously described [10]. In brief, CXCL12 was expressed as a His-tag fusion protein and purified from inclusion bodies. Bacterial cell pellets were sonicated and washed with deoxycholate following resuspension in 10 mM Tris pH 8.0 with 1 mM MgCl_2_, 200 µg DNAse, and Complete Protease Inhibitor Cocktail (EDTA-free) (Roche, Indianapolis, IN). Protein was then solubilized in 6 M Guanadine-HCl, 100 mM sodium phosphate, 10 mM Tris-Cl, pH 8.0, using a dounce homogenizer. CXCL12 was purified over a Ni-NTA column and refolded with Hampton Fold-It Buffer #8 (Hampton Research, Aliso Viejo, CA), then dialyzed and concentrated using Amicon Ultra centrifugal concentrators (MWCO = 5000). The His-tag was removed by cleaving with enterokinase (NEB, Ipswich, MA) at a 1∶100,000 molar ratio overnight at room temperature. CXCL12 was then purified by HPLC and the identity and purity was validated by ESI mass spectrometry. Transwell migration assays on Jurkat cells were used to validate functionality of the purified CXCL12.

### Migration assays

Transwell migration assays (Corning, Corning, NY) were performed on purified CLL B cells and B cells from healthy donors using inserts with a 6.5 mm diameter, 5.0 µm pore size. Cells were resuspended at 2.5×10^6^ cells/mL in RPMI+10%FBS and 100 µL of cell suspension was added to the inserts. CXCL12 was diluted over a concentration range of 0 nM to 500 nM in a 600 µL total volume of RPMI+10%FBS in the bottom wells. As a positive control and cell count reference, cells were added directly to the wells without inserts. Transwell migration was conducted for 2 h at 37°C/5%CO_2_. Cells that had migrated into the bottom wells were then collected and counted by flow cytometry on a FACSCalibur (BD Biosciences, San Jose, CA). Data was normalized to no chemokine control and percent migration was calculated from the positive reference control.

### Preparation of CLL lysates for proteomics

CLL cell lysates for phosphoproteomic analysis were prepared as previously described [10]. Briefly, 3×10^9^ total CLL PBMCs were washed with sterile PBS and resuspended at 1×10^7^ cells/mL in serum-free RPMI-1640 media. The CLL cell suspension was distributed evenly into five 15 cm plates (6×10^8^ cells/plate) (Corning Inc, Corning, NY) and cultured for 2 h at 37°C/5% CO_2_ prior to stimulation with CXCL12. CLL cells were then either unstimulated or stimulated for 3 min, 10 min, 30 min, or 60 min with 30 nM CXCL12. All plates were harvested at the same time with 3 mL ice cold cytoplasmic lysis buffer containing 10 mM HEPES, pH 7.9, 1.5 mM MgCl_2_, 10 mM KCl, 0.5 mM dithiothreitol (DTT) (Sigma, St. Louis, MO), Complete Protease Inhibitor Cocktail (Roche Diagnostics, Indianapolis, IN), and Halt Phosphatase Inhibitor Cocktail (Pierce, Rockford, IL) for 30 min on ice. Lysates were clarified by centrifugation at 20,000 rcf for 20 min at 4°C. The supernatants were distributed into protein LoBind Eppendorf tubes (Eppendorf, Westbury, NY) and stored at –80°C. The total protein concentration of the CLL lysates was determined using a BCA protein assay (Pierce, Rockford, IL).

### IMAC phosphopeptide enrichment

IMAC enrichment was performed as previously described [10]. Briefly, 2 mg of CLL lysates were denatured with 1% sodium dodecyl sulfate (SDS) (Fisher Scientific, Pittsburgh, PA), reduced with 10 mM DTT, and alkylated with iodoacetamide (Sigma, St. Louis, MO). Proteins were then precipitated with 50% ethanol/50% acetone/0.1% acetic acid (HAC). The pellets were resuspended in 6 M urea/0.1 M Tris, pH 8.0, and vortexed to solubilize the protein. The urea concentration was then diluted five-fold by addition of 50 mM Tris, pH 8.0 and protein was digested overnight at 37°C using sequencing-grade modified trypsin (Promega, Madison, WI) at a ratio of 1∶50 (trypsin:protein). Trypsin was inactivated by acidification of the digests with trifluoroacetic acid to 0.3 to 0.5% (v/v). Prior to IMAC enrichment, peptide mixtures were desalted with 50 mg Sep-pak C18 cartridges (Waters Corp, Milford, MA). IMAC beads were prepared by stripping Ni-NTA spin column resin (Qiagen, Valencia, CA) and recharging the beads with 100 mM FeCl_3_ (Fluka reagent, Sigma, St. Louis, MO). IMAC beads were then packed into gel loading tips with glass wool and conditioned with 25% Acetonitrile (ACN)/0.1% HAC. Nonspecific peptides were removed by washing twice with 30 µL of 25% ACN/0.1% HAC/0.1 M NaCl, twice with 0.1% HAC, and twice with 30 µL of Milli-Q H_2_O. Phosphopeptides were eluted with a total volume of 50 µL over three elutions with 1% phosphoric acid. All fractions were collected in protein LoBind Eppendorf tubes, speed-vac dried, and stored at –20°C until MS analysis. Analysis of IMAC washes and flow through by LC-MS/MS confirmed successful binding of phosphopeptides to the IMAC columns as less than 0.1% of these peptides were found to be phosphorylated, and the few that were detected were redundant with phosphopeptides identified in the IMAC enriched samples.

### Mass spectrometry and data processing

IMAC-enriched CLL peptides were resuspended in Milli-Q H_2_O +0.1%HAC and analyzed by reversed-phase, C18 capillary liquid chromatography and tandem mass spectrometry (LC-MS/MS) on a Thermo-Finnigan LTQ ion trap mass spectrometer. The capillary LC columns (∼17 cm) were pulled and packed in-house using deactivated fused silica (100 µm) (Agilent, Santa Clara, CA) as previously described [10]. Angiotensin II (Sigma-Aldrich, St. Louis, MO) was run after every two CLL sample runs as a control for column performance. The standard method used for all samples was as follows: 95% A/5% B (buffer A = 0.1% HAC in HPLC-grade Milli-Q H_2_O, buffer B = 0.1% HAC in HPLC-grade ACN) for 20 min, 60% A/40% B for 30 min, 20% A/80% B for 6 min, followed by a final washing step of 95% A/5% B for 30 min at 250 µl/min. A flow rate of 200 to 500 nL/min through the capillary column was achieved by splitting the flow of solvent before it reached the column. Samples were run in data-dependent mode in which the spectrometer performed one full MS scan followed by six MS/MS scans of the top six most intense ions in the parent spectrum with an *m/z* ranging from 400 to 2000. The dynamic exclusion list was varied in order to get a range of coverage and spectral counts. The standard dynamic exclusion list applied had a repeat count of 1, a repeat duration of 30 s, an exclusion size of 100, an exclusion duration of 180 s, and an exclusion mass width of 1.50. Other variations included a list of the top 25 peptides with a repeat duration of 60 s, an exclusion of the top 5 most abundant peptides, and a dynamic exclusion list turned off. The spray voltage was 1.8 kV. On average, the scan rate in this experiment ranged from four to eight scans per second.

A comprehensive phosphoproteomics data set from cells of one particular patient (CLL A) with unmutated IgHV and ZAP-70+ status (indicative of more aggressive disease) was collected from two separate triplicate runs and one duplicate run in order to obtain good coverage and number of spectra for comparison. Phosphoproteomics analyses of 4 additional CLL patients' cells (CLL B – E) (each run in single triplicate experiments) were also performed to ensure reproducibility between different patient cells and stimulations. CLL B, D and E also had more aggressive (high ZAP-70) characteristics (high ZAP-70) while CLL C was of the indolent (low ZAP-70) subgroup. Following data collection, RAW data files were converted to mzXML data files using the program ReAdW (http://tools.proteomecenter.org/ReAdW.php). Data analysis of the MS/MS spectra was performed using the open-access database search tool, InsPecT [11], [12], with the UniProt human database, the UniProt shuffled human decoy database as well as common contaminants databases (e.g. keratin). Peptide sequencing searches were defined for tryptic cleavage restraints and to allow for modification of up to two phosphorylation sites (Ser, Thr, or Tyr) on a peptide. Spectra were sorted by *p*-values according to the InsPecT scoring function and the target decoy database was used as a measure of the overall quality of MS/MS data. Peptides smaller than 7 amino acids and peptides with more than one missed cleavage were excluded from analysis. Peptides with a false discovery rate (FDR) of less than 1–2% were manually validated for positive identification.

### Western blots and antibody reagents

For western blot analysis, patient CLL cells used for phosphoproteomics as well as additional patient's cells were used. Purified CLL B cells were cultured in serum-free RPMI at 1×10^7^ cells/mL for 2 h at 37°C/5%CO_2_ and then stimulated with 30 nM CXCL12 over an hour time course (unstimulated, 3, 10, 30 and 60 min) or for 4 h, 10 h, and 24 h for PDCD4 degradation analysis. For inhibitor studies, CLL cells were pre-treated with 40 µM AMD3100 (Sigma) or 200 ng/ml Pertussis toxin (List Biological Laboratories) for 1 h prior to stimulation with CXCL12. Coculture of CLL cells with NLCs was performed as previously described [3], [9] and then the CLL cells were collected and centrifuged for harvest. Cells were lysed on ice for 30 min in Ripa buffer (10 mM Tris pH 7.4, 150 mM NaCl, 1% Triton X, 0.1% Na-Deoxycholate, 0.1%SDS and 5 mM EDTA) containing Complete Protease Inhibitor Cocktail (Roche, Palo Alto, CA) and Halt Phosphatase Inhibitors (Pierce). Lysates were clarified by centrifugation at 20,000 rcf for 10 min at 4°C. A BCA protein assay (Pierce) was performed to determine total protein concentration and 20 µg of total protein was loaded into each well of a Criterion 4–12% Bis-Tris gel and run with the XT MES buffer system (Bio-Rad, Hercules, CA). Gels were transferred onto PVDF membranes (Bio-Rad), blocked with 5% milk-TBST, and incubated overnight at 4°C with primary antibodies. Blots were washed 3 times for 10 min with Tris Buffered Saline +0.1% Tween (TBST) and then incubated for 1 h at room temperature with secondary antibodies conjugated to HRP, washed again 3 times with TBST and then developed with Amersham ECL-plus (GE-healthcare) or SuperSignal West femto-sensitivity reagent (Pierce). Blots were stripped with Restore western blot stripping solution (Pierce) for 10 min at room temperature and then re-probed with other antibodies and/or β-actin as a loading control. Primary antibodies were diluted into 5% BSA-TBST at recommended concentrations. Phospho-PDCD4 and PDCD4 antibodies were obtained from Rockland Immunochemicals. Phospho-p38, phospho-HSP27, HSP27, phospho-S6K, and β-actin were obtained from Cell Signaling Technology. Densitometry analysis was performed using ImageJ software (NIH) and normalized to β-actin loading controls.

### Flow cytometry

CLL B cells or normal B cells were purified for flow cytometry analysis. Cells were washed and resuspended in a 0.5% bovine serum albumin (BSA) (Sigma) in Phosphate Buffered Saline (PBS) solution and stained for CXCR7 expression using an APC-conjugated antibody (clone 11G8) (R&D systems) or an APC-conjugated IgG1 isotype control according to manufacturer's protocol (R&D systems). Flow cytometry data was collected on a FACSCalibur cytometer (BD Biosciences) and analyzed using FlowJo software.

## Results

### Normal B cells migrate with higher efficacy and potency to CXCL12 than CLL B cells, despite having lower levels of CXCR4

Chemokines, including CXCL12, are best known for their role in directing cell migration, but it is well established that they can also induce cell survival and proliferation [8]. This observation can be rationalized by the fact that some of the major pathways involved in cell migration (e.g. PI3K/Akt and Raf/MEK/ERK), are also important for survival and proliferation signaling [13]. However, little is known regarding the extent to which there is overlap or divergence of the upstream and downstream effectors of these pathways in the context of migration versus survival/proliferation. Since it has been established that CLL cells have up-regulated expression of CXCR4 compared to normal B cells, and that CXCL12 stimulation of CLL cells activates Akt and ERK1/2 pathways, transwell migration assays were performed on purified CLL B cells and normal B cells to compare their ability to migrate towards CXCL12 [5], [8], [9].

Surprisingly, the normal B cells showed a significantly greater (p<0.0001) ability to migrate to CXCL12, with respect to both efficacy (4.5+/−1.2% migration in normal vs. 0.85+/−0.48% migration in CLL cells) and potency (∼10 nM in normal vs ∼50 nM in CLL cells) (Figure 2). Although it was expected that the CLL cells would have the stronger migratory response due to higher CXCR4 expression, these results are consistent with previously published observations that showed weak migration of CLL cells to CXCL12 compared to a much more robust response to the CCR7 ligands, CCL19 and CCL21 [14], [15]. These data suggest that the downstream effects of CXCR4 may be redirected for survival rather than migration, and led us to consider what other pathways or downstream targets of Akt or ERK1/2 might be activated to bias the CXCL12 signaling response towards survival. Taking a global approach to this question, we performed mass spectrometry-based phosphoproteomics analysis of unstimulated and CXCL12-stimulated CLL cells.

### Characterization of phosphopeptides/phosphoproteins in CXCL12-stimulated CLL cells via mass spectrometry

Fresh PBMCs from 5 CLL patients were stimulated over an hour time course with CXCL12, and lysates were generated for IMAC enrichment and LC-MS/MS analysis. Multiple (3 separate duplicate or triplicate experiments with variable acquisition methods) phosphoproteomics data sets were acquired on lysates from the cells of a patient with ZAP-70+ aggressive CLL, referred to as “CLL A”, in order to ensure good coverage of the proteomic space. Smaller phosphoproteomics data sets (single triplicate experiments) were collected from cells of 4 additional patients (CLL B to E) for comparison.

**Figure 3:**
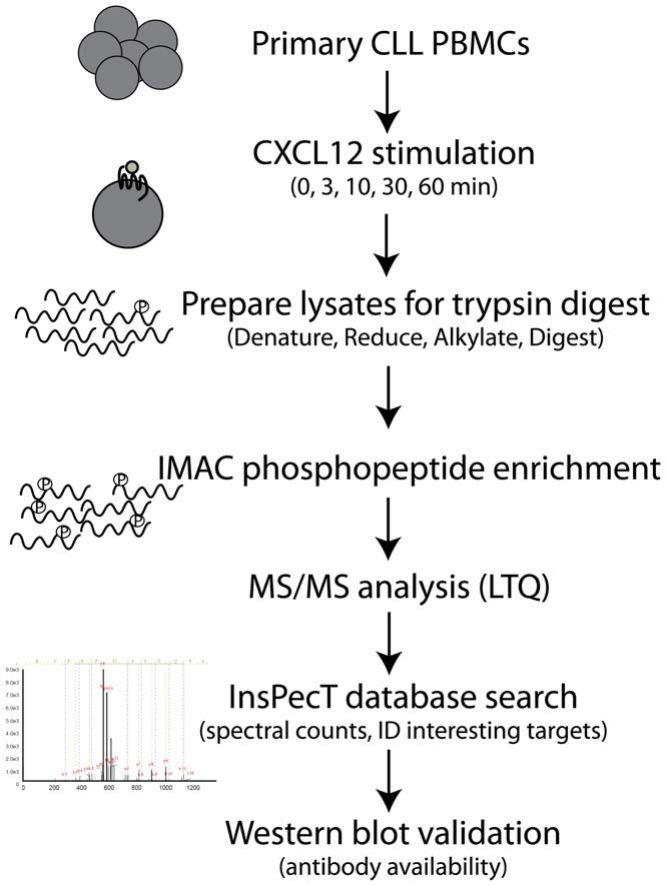
Flow Chart of CLL Phosphoproteomics Analysis. Lysates were prepared from primary CLL B cells that had been stimulated over an hour time course with 30 nM CXCL12. Lysates were denatured, reduced and alkylated in preparation for trypsin digest. Tryptic peptides were then enriched for phosphopeptides by IMAC and LC-MS/MS was performed. Data was analyzed using the InsPecT database search algorithm for phosphorylations on Ser, Thr, or Tyr. A decoy database and manual validation of the spectra were used as quality control. Spectral count comparisons were made as a qualitative assessment of CXCL12 stimulation response and interesting target proteins were selected for follow-up studies if antibodies were available.

Protein lysates from the CLL cells were trypsin digested and enriched for phosphopeptides via immobilized metal affinity chromatography (IMAC) to yield a highly enriched population of phosphorylated peptides. Phosphopeptides were analyzed by LC-MS/MS using a linear iontrap (LTQ) mass spectrometer. Data was processed using the InsPecT database search algorithm and the spectra were manually inspected for validation (see flow diagram Figure 3).

Over 10,000 spectra were collected from the combined phosphoproteomics analysis of unstimulated as well as stimulated CLL cells, which was comprised of 1470 phosphopeptides (>1200 unique phosphosites) from 696 phosphoproteins (Table S1). In general, a >30% enrichment of annotated phosphopeptides was achieved from our IMAC procedure, which is on par with other phosphopeptide enrichment studies of mammalian cells [16].

**Figure 4:**
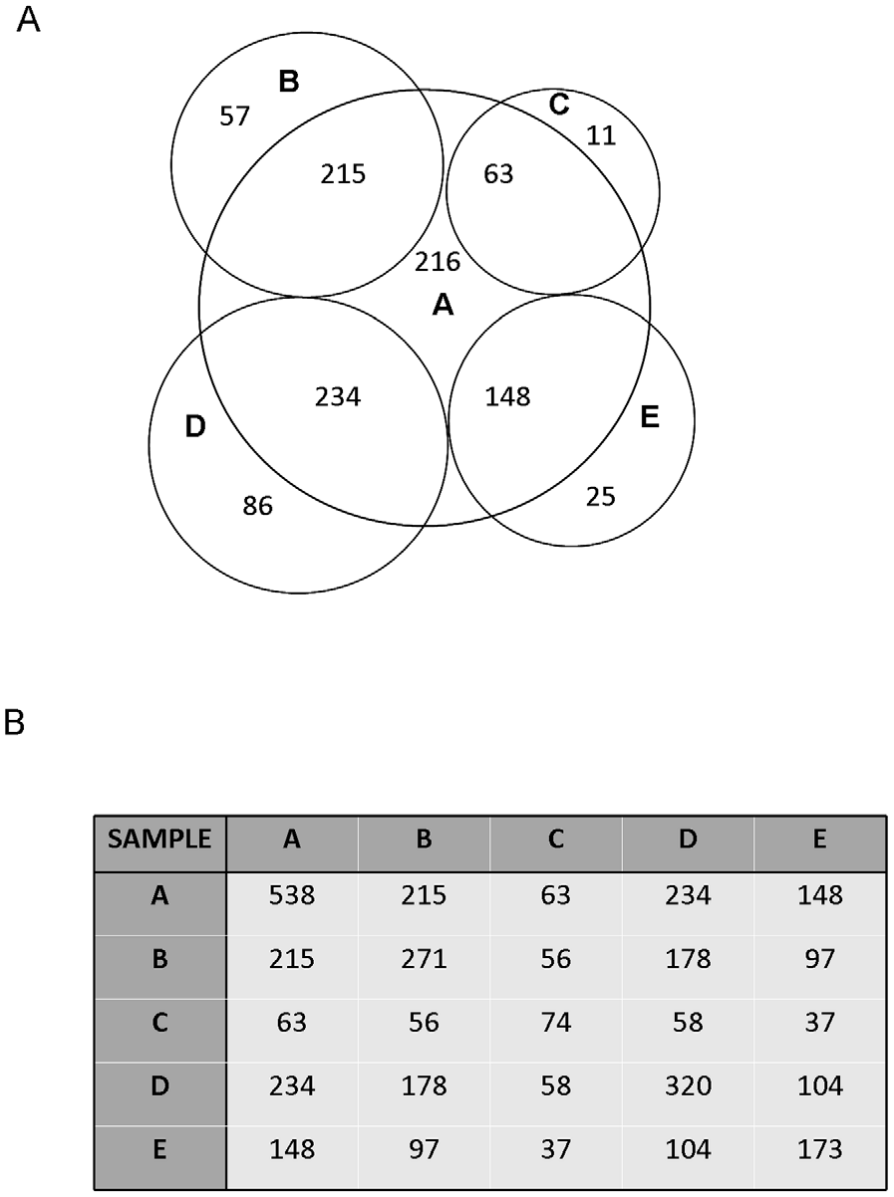
Overlap in Phosphoprotein Identification between CLL cells from Different Patients. A) Venn diagram illustrating the degree of overlap between the phosphoproteins identified in CLL A compared to the phosphoproteins identified in CLL B, C and D. B) Matrix table outlining the number of overlapping phosphoproteins identified for CLL A, B, C and D.

To ensure that good coverage of the CLL A data set was obtained, a comparison was made of the overlapping phosphoproteins identified in the CLL A data sets and the smaller CLL B - E data sets. 538 of the 696 total phosphoproteins (>77%) identified in the CLL A data sets were also identified in data sets B - E, suggesting detection of the majority of phosphopeptides detectable by these methods. Also, many of the non-overlapping phosphoproteins identified in CLL B to E but not in CLL A were isoform variants of phosphoproteins detected in CLL A. Figure 4 depicts the overlap of CLL B - E with CLL A (Figure 4A) and the matrix shows overlap between all of the CLL samples (Figure 4B).

### Identification of phosphoproteins with prior correlations to CLL and other leukemias

Adding confidence to our phosphoproteomics results, a number of hematopoietic-specific phosphoproteins as well as phosphoproteins with prior implications in CLL and other leukemias were identified. Table 1 highlights 9 of the phosphoproteins detected with previous links to CLL/leukemia, including Hematopoietic cell-specific Lyn substrate (Hcls1) and SH2-containing inositol phosphatase-1 (SHIP-1). The phosphorylation status of both Hcls1 and SHIP-1 have been shown to correlate with disease aggressiveness and shorter mean survival [17], [18], [19], consistent with the aggressive characteristics of CLL A.

**Table 1:** Phosphoproteins identified by LC-MS/MS analysis with prior implications in CLL/leukemia disease.

Protein	Gi Accession	Implications in CLL/leukemia	References
B cell novel protein 1 (BCNP1)	31542207	Overexpressed in CLL and other B cell malignancies compared to normal B cells	Boyd et al 2003
Formin-like 1 (FMNL1)	33356148	Akt interacting partner found to be overexpressed in CLL	Favaro et al 2003
Hematopoietic cell-specific Lyn substrate (Hcls1 or HS1)	4885405	Phosphorylation correlated with shorter mean patient survival time in CLL	Scielzo et al 2005
HSP-90 alpha	92859630	Stabilizes Akt and ZAP-70 signaling in CLL, highly activated in CLL, and role in CLL survival	Castro et al 2005
Lyn (Yamaguchi sarcoma viral (v-yes-1))	4505055	Overexpressed in CLL compared to normal B cells, potential anti-apoptotic function in CLL	Contri et al 2005
Minichromosome maintenance protein 2 (Mcm2)	33356547	Role in DNA replication, marker for proliferation and prognosis in B-cell lymphoma	Obermann et al 2005
promyelocytic leukemia protein (PLZF)	4505903	Correlates with apoptotic resistance, higher expression of PLZF associated with lower CLL patient survival	Parrado et al 2000
SH2 containing inositol phosphatase 1 (SHIP-1)	64085167	Phosphorylation status segregated with ZAP-70, correlated with aggressive disease in CLL	Gabelloni et al 2008
Stathmin 1 (oncoprotein 18)	44890052	Overexpressed in acute leukemia cells compared to normal lymphocytes, involved in cell growth and proliferation	Melhem et al 1991

Table listing select proteins identified by LC-MS/MS with prior implications in CLL or other related leukemias along with the GI accession number, a brief description of biological implications, and references [17], [19]–[20], [22], [41]–[45].

Also included in Table 1 are phosphoproteins which have been linked to CLL but not necessarily phosphorylation status, including HSP90, B cell novel protein 1, promyelocitic leukemia protein, and formin-like 1 (FMNL1) [20], [21], [22]. These proteins have general implications in CLL, but whether differences in phosphorylation status affect the activity or function in disease is unclear. A few additional proteins including minichromosome maintenance protein 2 and stathmin 1 have been linked to disease progression of other leukemias, but not directly to CLL and thus warrant further investigation in CLL. Many of these phosphoproteins did not appear to exhibit changes in phosphorylation in response to CXCL12 or spectral numbers were too low to make an assessment of stimulation response, but HSP-90 and Mcm2 could be potential phosphorylation targets and are thus also highlighted as proteins of interest in Table 2.

**Table 2:** Select phosphoproteins from phosphoproteomics analysis with spectral count numbers and known functions.

Adenylyl cyclase-associated protein (CAP1)	5453595	5; 8; 14; 17; 12	Invovled in cAMP pathway, overexpressed in pancreatic cancer, correlated with poor prognosis	Yamazaki et al 2009
Heat shock protein 27 kDa (HSP27)	7706687	1; 10; 13; 7; 7	Downstream target of p38-MAPKAPK2 pathway; involvement in IkB degradation, protection from apoptosis, sequestering and inhibiting cytochrome c release, interacts with Akt	Parcellier et al 2003; Garrido et al 2006
Heterogeneous nuclear ribonuclear proteins (U, D, A1)	U (14141161), D (14110414), A1 (45044445)	U (0; 14; 11; 6; 7), D (3; 21; 25; 20; 14), A1 (13; 33;16; 40; 17)	Nucleocytoplamic shuttling proteins that shuttle mRNAs from site of transcription to start of translation; hnRNPA1 levels are increased in chronic myelogenous leukemia (CML), implications in apoptotic resistance and tumorigenesis; potentially regulated by phosphorylation by various MAPKs	Lervolino et al 2002; Eiring et al 2008; Van der Houven van Oordt et al 2000
HSP90-alpha	92859630	2; 24; 18; 21; 12	Stabilizes PI3K and Akt, pro-proliferative and tumorigenic effects, important for Jak-STAT signaling, Implicated with ZAP-70 stability and signaling in aggressive CLL, less known regarding phosphorylation	Castro et al 2005; Fujita et al 2002; Sato et al 2000; Schoof et al 2009
L-Plastin (lymphocyte cytosolic protein 1)	4504965	0; 4; 9; 1; 1	Actin binding protein expressed in hematopoietic lineage cells as well as malignant cells of non-hematopoietic origin; important for cell polarization and motility	Lin et al 1993; Morley et al 2010
Lymphocyte specific protein 1 (LSP1) (S252)	61742789	1; 9; 11; 5; 8	Downstream target of p38-MAPKAPK2 pathway, PKC, GSK3; F-actin binding protein involved in chemotaxis	Wu et al 2007
Minichromosome maintenance protein 2 (Mcm2)	33356547	2; 7; 2; 4; 2	Role in DNA replication, marker for proliferation and prognosis in B-cell lymphoma	Obermann et al 2005
Programmed cell death factor 4 (PDCD4)	21735596	5; 11; 10; 9; 14	Inhibition of AP1 transcription and eiF4A translational activity, phosphorylation by Akt and p70 S6K is inhibitory and promotes its degradation	Yang et al 2001; Yang et al 2003; Palamarchuk et al 2005; Lankat-Buttgereit et al 2009
serine/arginine repetitive matrix 1 (SRm160)	42542379	15; 57; 57; 40; 30	RNA splicing coactivator; regulates CD44 alternative splicing with potential role in tumor cell invasion	Cheng and Sharp 2006
Small acidic protein	7657234	1; 18; 10; 14; 6	Very little information available, unknown function	
Splicing factor 1 (SF)	42544125	0; 14; 13; 21; 13	Regulates premesseger RNA splicing and gene transactivation and including that of b-catenin/TCF4 complex. Phosphorylated by protein kinase KIS enhances binding to U2AF65	Shitashige et al 2007; Manceau et al 2006
UV excision repair protein RAD23 homolog A (RAD23A, hHR23)	4826964	0; 19; 10; 12; 3	Involved in nucleotide excision repair, recognition of DNA damage; implicated in p53 degradation; phosphorylation function unknown	Glockzin et al 2003

Spectral count numbers for select phosphoproteins of interest are presented for each CXCL12 (30 nM) stimulation time point (0; 3; 10; 30; 60 min). A brief description of known functions and corresponding references are also provided [21]–[24], [26], [28]–[29], [43], [46]–[60].

### Identification of novel downstream targets of CXCL12/CXCR4 signaling in CLL

To semi-quantitatively assess whether phosphorylation of some of the proteins is a consequence of CXCL12 stimulation, spectral counts from the mass spectrometry runs on the stimulated samples off CLL A were compared to those from unstimulated samples. Select candidate targets of CXCL12-induced phosphorylation are reported in Table 2 along with their associated spectral counts. A number of known targets of survival signaling pathways including programmed cell death factor 4 (PDCD4) and heat shock protein 27 (HSP27) were identified as having more spectral counts in the stimulated versus unstimulated samples, and were selected for validation by western blot and further analysis, discussed in detail later. Validation was performed on lysates from CLL cells used in phosphoproteomics analysis (lettered CLL A to E) as well as additional patient cells not examined by phosphoproteomics (CLL1, CLL2, etc) in order to determine consistency of these responses across different patients, since CLL is a heterogeneous disease [1].

Some of the phosphoproteins identified in this study have been previously implicated in cancer malignancy such as Mcm2 and adenylyl cyclase associated protein (CAP1), while little information is available on some of the other potential targets including small acidic protein (Table 2). Although two of the proteins (PDCD4 and HSP27) are validated herein as targets of CXCL12-signaling in CLL, the remaining phosphoproteins, while beyond the scope of this work, pose interesting targets for future investigations.

### CXCL12 induces the phosphorylation and degradation of PDCD4

**Figure 5:**
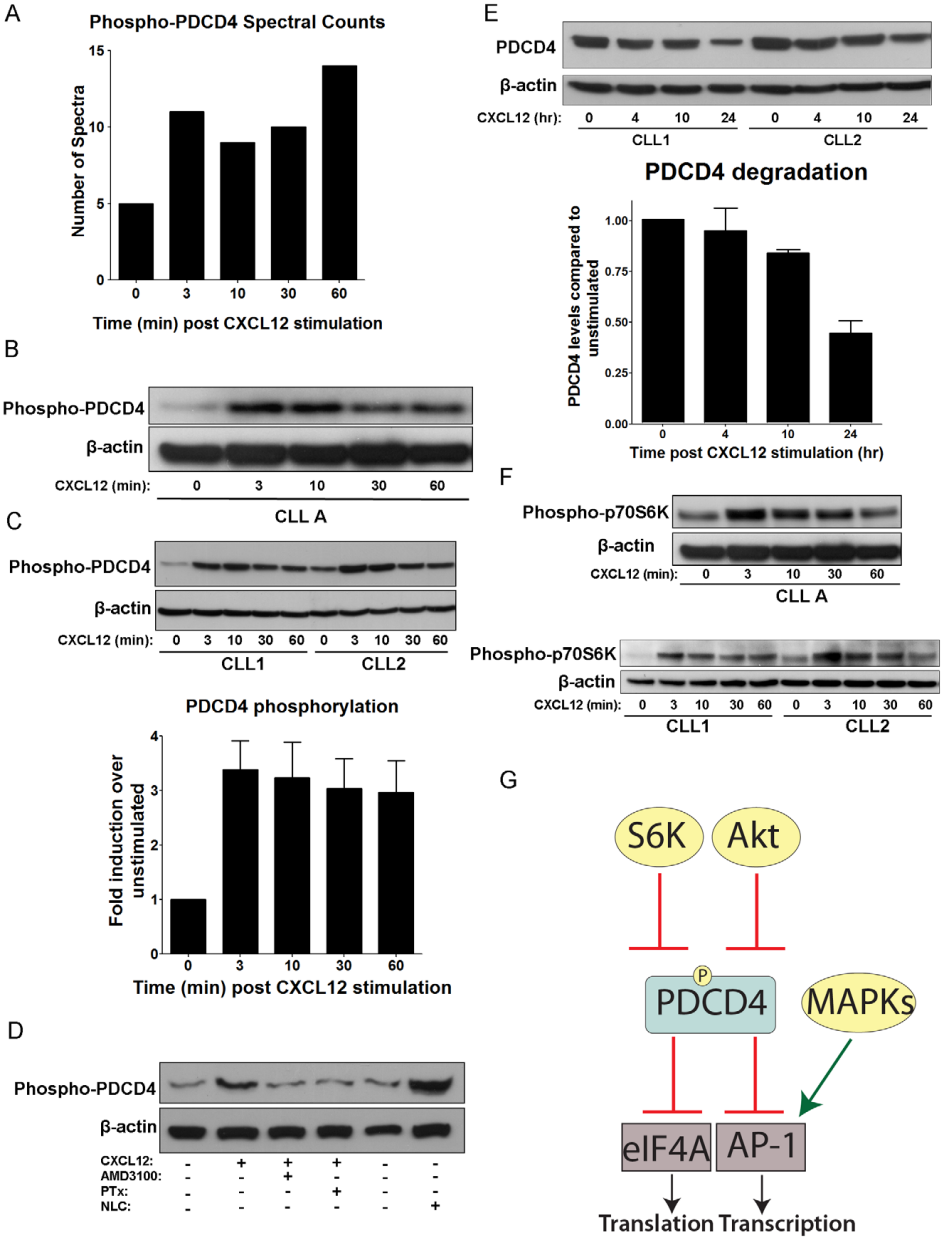
CXCL12 Induces Phosphorylation of PDCD4 at Ser457. A) Bar graph depicting the spectral counts of PDCD4 phosphopeptides observed in the LC-MS/MS analysis at time points of CXCL12 stimulation. B) Western blot of PDCD4 phosphorylation over time course of 0 to 60 min CXCL12 stimulation (30 nM) from CLL A patient cells. β-actin served as a loading control. C) Top panel: Representative western blot of PDCD4 phosphorylation in CLL cells from 2 different CLL patients not used in LC-MS/MS analysis over 30 nM CXCL12 stimulation time course. β-actin served as a loading control. Bottom panel: Densitometry analysis of PDCD4 phosphorylation levels CXCL12 stimulation (30 nM) time points relative to unstimulated controls and averaged from 10 separate CLL patient cells. Error bars represent standard error of the mean (SEM). D) Western blot of PDCD4 phosphorylation in unstimulated/untreated CLL cells or 3 min CXCL12 stimulations (30 nM) in the presence (+) or absence (−) of preincubation (1 h) with AMD3100 (40 µM) or Pertussis toxin (PTx) (200 ng/ml). NLC lysate represents CLL cells cultured in presence of NLCs with no further stimulation or treatment. CLL cells were removed from the adherent NLCs and lysed. β-actin served as a loading control. E) Top panel: Representative western blot detecting total levels of PDCD4 in CLL cells following 0, 4, 10 or 24 h of 30 nM CXCL12 stimulation. β-actin served as a loading control. Bottom panel: Bar graph quantifying total PDCD4 levels over 24 h time course of 30 nM CXCL12 stimulation compared to 0 h unstimulated controls and normalized to β-actin levels by densitometry analysis of western blots. Data represented are mean +/− SD of 3 separate CLL patients' cells. F) Western blot stripped and reprobed from Figure 4B for p70S6K phosphorylation (Thr389) over time course of 30 nM CXCL12 stimulation from CLL A patient cells (Top panel) and 2 other representative CLL patients' cells (bottom panel). β-actin served as a loading control. G) Diagram of PDCD4 signaling showing known upstream regulators as well as downstream targets. Akt and p70S6K are known to phosphorylate PDCD4, thereby inhibiting its function in repressing eIF4A translational activity and AP-1 transcription.

PDCD4 is one of the phosphoproteins that appeared to be induced by CXCL12 stimulation based on spectral counts (Figure 5A and Table 2). It is a known tumor suppressor, and downstream phosphorylation target of Akt, which is known to be activated by CXCL12 in CLL cells [9]. A phospho-specific antibody is also commercially available, making it attractive for follow-up studies [23], [24]. As a tumor suppressor protein, PDCD4 has been implicated in a number of cancers where it is often inhibited and/or downregulated, disrupting its ability to inhibit eIF4A translational and AP-1 transcriptional activity, processes that are important for cell growth and survival (Figure 5G). Phosphorylation of PDCD4 is known to occur by Akt and p70 S6Kinase (p70S6K) which inhibits its activity and leads to its ubiquitination and proteosomal degradation [23], [24], [25], [26].

Three separate phosphopeptides from PDCD4 were detected from our analysis: R.FVSpEGDGGR.V (Ser457), R.SGLTVPTSpPK.G (Ser94) and R.DSGRGDSpVSDSGSDALR.S (Ser76) (Figure S1). The R.FVSpEGDGGR.V phosphopeptide corresponds to Ser457 phosphorylation, a site known to be phosphorylated by Akt [23]. Therefore, multiple CLL patient samples were examined for PDCD4 phosphorylation in response to CXCL12 by western blot. An increase in phosphorylation of PDCD4 at Ser457 was observed upon CXCL12 stimulation in CLL A cells (Figure 5B), as well as all 9 additional CLL patient cells examined (representative western blot in Figure 5C). Increases in PDCD4 phosphorylation levels was variable between patients and ranged from 1.7-fold to 7.4-fold and averaged to approximately 3.4-fold (n = 10), as quantified by densitometry analysis of western blots (Figure 5C). Although variability was noted, this variation did not cluster according to disease aggressiveness.

As a control to ensure that the phosphorylation was dependent on CXCL12/CXCR4 signaling and to determine if the effects were dependent on signaling through the G-protein, Gi, CLL cells were pretreated with the small molecule CXCR4 antagonist, AMD3100, or the Gi-inhibitor, pertussis toxin (PTx) prior to a 3 min stimulation with CXCL12. As shown in Figure 5D, both AMD3100 and PTx completely abrogated phosphorylation suggesting it is CXCR4 and G-protein signaling dependent. Furthermore, to ensure that phosphorylation of PDCD4 has relevance in a more physiological context, the levels of PDCD4 phosphorylation were examined in CLL cells that had been cultured with NLCs (+) compared to those without NLCs (−). As with CXCL12 stimulation, the coculture of CLL cells with NLCs led to an increase in the phosphorylated levels of PDCD4 (Figure 5D).

Since the phosphorylation of PDCD4 is known to lead to its ubiquitination and degradation [23], [25], we examined levels of total PDCD4 over a 24 h time period (0, 4, 10 and 24 h) following CXCL12 stimulation. As shown in Figure 5E, 24 h post-stimulation resulted in PDCD4 degradation to ∼45% of starting levels.

Additionally, although it is well established that Akt is phosphorylated downstream of CXCL12 signaling in CLL cells [9], it has not been established whether p70S6K, another kinase known to phosphorylate PDCD4 leading to its ubiquitination and degradation, is activated in CLL cells by CXCL12. Western blot analysis revealed that phosphorylation and thus activation of p70S6K (Thr389) was induced by CXCL12-stimulation in the CLL cells (Figure 5F).

### HSP27 expression and phosphorylation is variable in CLL cells

**Figure 6:**
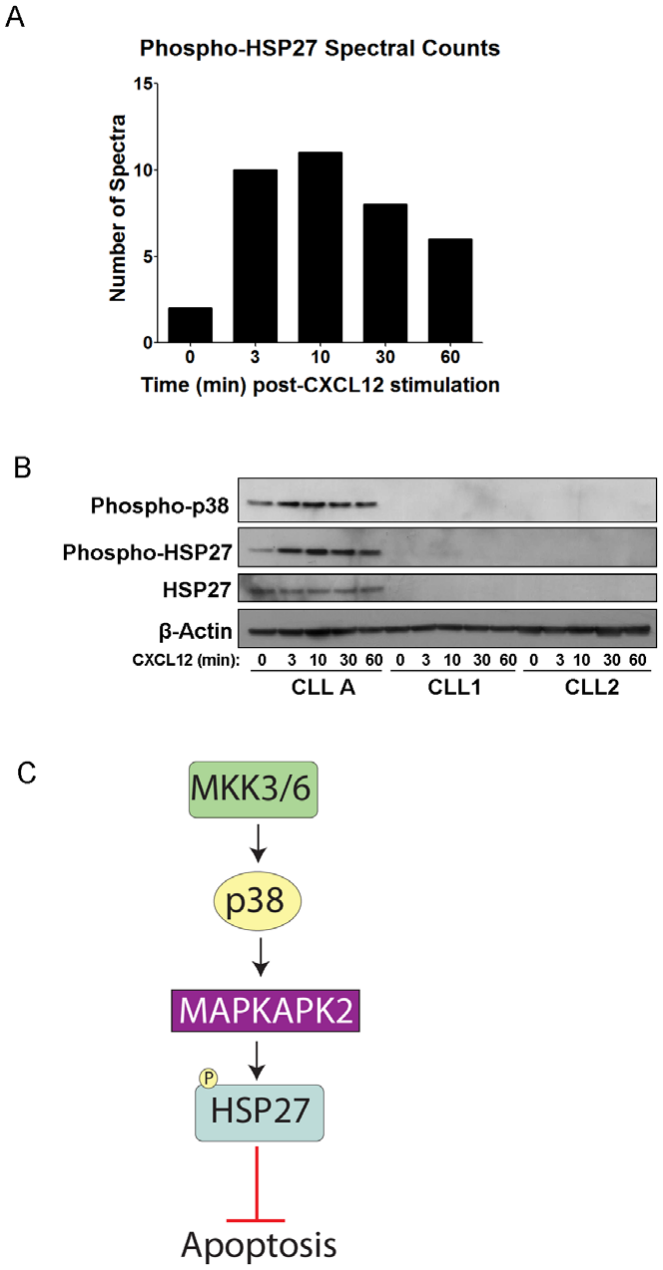
Phosphorylation of HSP27 in Subset of CLL Patients. A) Bar graph depicting the spectral counts of HSP27 phosphopeptides (Ser82) observed in the LC-MS/MS analysis after CXCL12 stimulation. B) Western blot detecting phosphorylation of HSP27 and the upstream p38-MAPK, and total HSP27 over time course of 0 to 60 min CXCL12 stimulation (30 nM) from CLL A patient cells and 2 other representative CLL patients' cells. β-actin was run as a loading control. C) Signaling diagram of HSP27, which can protect from apoptosis, and its upstream regulation by p38-MAPK and MAPKAPK2.

The other phosphoprotein investigated further was HSP27 (phosphopeptide: R.QLSphosSGVEIR.H, Ser82) (mass spectrum shown in Figure S2). HSP27 was also selected due to spectral counts indicative of phosphorylation upon CXCL12 activation of CXCR4 (Figure 6A), its implications in cancer and protection from apoptosis, and the availability of a phospho-specific antibody at the Ser82 phosphorylation site [27], [28], [29]. Additionally, HSP27 is an interesting target since it is downstream of p38-MAPK signaling [27] which has not received much attention in association with CLL and therefore represents a pathway with potentially novel implications in CLL survival.

Interestingly, while PDCD4 was found to be a common target of CXCL12 signaling in all CLL samples examined, phospho-HSP27 and total HSP27 protein were only detectable by western blot in a subset of ∼25% (3 out of 12) of CLL patients examined (representative western blot Figure 6B). Nevertheless, HSP27 was indeed present and did exhibit an increase in phosphorylation upon CXCL12 stimulation in the CLL A patient samples from which the phosphoproteomics data was collected (Figure 6B). Since p38-MAPK is known to be upstream of HSP27 phosphorylation, we also examined p38 phosphorylation among the CLL patient samples (Figure 6C); correspondingly, we observed detectable p38 phosphorylation only in the samples that also exhibited the HSP27 phosphorylation (CLL A, Figure 6B). Although no common factor could be determined among the patients examined with detectable HSP27, a larger sample size might identify common features of these cells and determine whether HSP27 is influencing the survival of this subset of CLL patients.

## Discussion

CLL is the most common leukemia in the Western world [1]. The accumulation of CLL B cells is believed to result from low rates of precursor cell proliferation and via recruitment of accessory cells that create a supportive microenvironment by producing factors that foster CLL survival [1], [2]. The chemokine, CXCL12, is one of the cytokines produced by cells in the microenvironment that enhances CLL survival *in vitro* and likely *in vivo* [9]. Although chemokines are best known for their role as chemoattractants, we show here that CLL cells are much less capable of migrating to CXCL12 compared to CLL B cells, despite an upregulation of CXCR4 on the CLL cells [5]. While the low levels of migration may still play a role *in vivo* [30], it is evident that the CXCL12/CXCR4 axis is also networked into pathways involved in survival. In contrast, CXCR7, the other receptor of CXCL12, is not expressed on the surface of CLL cells although it is expressed on normal B cells [6]. Thus, while there is overlap in signaling pathways activated by CXCL12 in CLL cells and normal B cells, the differences in migration and CXCR7 expression, and the potential bias towards survival in CLL cells, suggest significant differences in the role that the CXCL12/CXCR4 axis plays in the context of the normal and pathological cells.

Phosphoproteomics analysis of CXCL12-stimulated CLL cells was performed in an effort to determine potential downstream signaling targets that could contribute to the survival and malignancy of CLL cells. As these are precious non-renewable primary patient cells, the intent of our phosphoproteomics approach was to generate hypotheses rather than an exhaustive analysis of the CLL phosphoproteome. Therefore, while it would be ideal to use a number of phospho-enrichment strategies in addition to IMAC (e.g. TiO_2_) and to employ additional liquid chromatography separation steps besides C18 (e.g. hydrophilic interaction liquid chromatography (HILIC)) to expand the number of phosphoproteins identified, we focused our efforts on well established methods [10], [31]. Along these lines, the use of quantitative phosphoproteomics strategies is limited since these cells do not replicate and cannot be cultured long term. Thus, stable isotope labeling of amino acids in cell culture (SILAC) is not possible [32]. Post-digest labeling with iTRAQ or ICAT isotopic labels [32] is also difficult due to limited sample availability, limitations in instrumentation (one-third rule with the LTQ spectrometer restricting detection of the labels in the low molecular weight range) [33], and the labile nature of phosphates and labels which causes reduced fragmentation and detection in MS/MS spectra [34]. While understanding its limitations, spectral counting was employed as a semi-quantitative assessment of the CXCL12-stimulation responses [35] and several candidates were followed up by western blot validation. In addition to the above examples with PDCD4 and HSP27, which showed that the spectral counting provides a relatively good approximation of stimulation response, spectral counts reflecting fairly even levels of phosphorylated p21-activated kinase (PAK2), another target of the PI3K pathway, was also confirmed by western blot in all six patient cells probed for phospho-PAK2 (Ser141) (Figure S3).

Through this phosphoproteomics approach, we were able to confidently identify close to 700 phosphoproteins in the CLL samples, including numerous proteins previously implicated in CLL disease. Additionally, we identified many proteins that appear to exhibit changes in phosphorylation levels in response to CXCL12. This data led to the identification and validation of several previously unknown phosphorylation targets of CXCL12 signaling. Typical approaches (e.g. western blot) for investigating signaling in response to stimuli require *a priori* knowledge of specific targets and the availability of phospho-specific antibodies, which limits the ability to globally assess cellular signaling events. Furthermore, validation studies of CLL cells are difficult since their viability in culture is limited. They are also difficult to manipulate through transfection and transduction since they do not divide in culture or infect well (e.g. they require high MOI and/or pre-activation of cells with CD40L and IL-4 [36] which could complicate interpretation of signaling analysis). Therefore, this mass-spectrometry-based approach seemed the most effective method for gaining new insight into the function of CXCL12 on CLL cell survival and possibly disease aggressiveness. Although not done in this study, comprehensive MS analysis of many patients may help to distinguish variations between patients, and with disease stratification and the identification of judicious therapeutic targets.

To our knowledge, this is the first report demonstrating that PDCD4 is a phosphorylation target downstream of CXCL12 signaling in CLL or other cell types. This finding is exciting due to the established role of PDCD4 as a tumor suppressor and as a substrate of Akt [23], [25]. Although little is known regarding the function of two of the phosphorylation sites of PDCD4 (Ser94 and Ser76) identified from the LC-MS/MS analysis, phosphorylation at Ser457 near the C-terminus of the protein is a well-established site with known functional implications. Phosphorylation at Ser457 by Akt has been shown to result in nuclear translocation of PDCD4 and a decrease in its ability to inhibit AP-1-mediated transcription and eIF4A-mediated translation [24], [26]; although Ser67 phosphorylation was not directly identified in our LC-MS/MS analysis, it is likely that this sight is also phosphorylated in response to CXCL12 stimulation since PDCD4 degradation was observed following stimulation [23], [25]. In combination, these effects may reduce the growth regulating/tumor-suppressor capacity of PDCD4, thereby contributing to CLL cell survival and the malignancy phenotype. Validation of PDCD4 phosphorylation also led to the identification of p70S6K phosphorylation and activation downstream of CXCL12 signaling in CLL cells.

Based on the phosphoproteomics analysis, HSP27 appeared to be another promising phosphorylation target of CXCL12-signaling. HSP27 and several other HSPs have received attention in the context of cancer due to their cytoprotective/anti-apoptotic functions. Specifically, HSP27 indirectly inhibits cytochrome c release and caspase activation and it sequesters cytosolic cytochrome c. It also promotes degradation of the inhibitor of NF-kB (IkB) and p27kip, and interacts with and supports the activity of Akt under stressful conditions, all leading to protection from apoptosis [29], [37]. We identified the presence of phosphorylated and total HSP27 protein and its upstream activator p38-MAPK in a subset (∼25%) of cell samples from different CLL patients. Of note, the observed variability in signaling between different CLL patient cells highlights the underlying heterogeneity of the disease. Furthermore, it serves as a reminder of how different parameters including patient differences (age and gender), variations in clinical course such as aggressiveness, stage and prognosis, and different treatments (e.g. chemotherapy, gene therapy, etc) may alter how the cells respond to different stimuli. Such variability is not unprecedented as Messmer *et al* (submitted manuscript) have demonstrated differences in CXCL12-mediated MEK and ERK activation in different ZAP-70 subgroups of CLL, and Montresor *et al* demonstrated differences in CXCL12-mediated lymphocyte function-associated antigen-1 (LFA-1) activation in normal B cells compared to CLL B cells and amongst the cells of different CLL patients [38]. Thus, it is reasonable to expect that different patients will exhibit different responses to stimuli, whether it is a survival stimulus from the microenvironment or a therapeutic agent used to treat the disease.

These results showing patient variability in HSP27 expression emphasize the strength of utilizing primary cells for understanding disease pathogenesis as opposed to cell lines, which are much more homogenous, but can be less insightful and sometimes misleading. While there was considerable overlap in the phosphoproteins identified from LC-MS/MS analysis between different patients (CLL A – E), more comprehensive MS analysis of multiple patients may help to distinguish variations in signaling responses between patients. Along these lines, since HSP27 is often induced following stressful cellular events such as treatment with chemotherapeutics, its induction in certain patients could reflect a response to treatment. For example, lymphoma cells which did not express Hsp27 were sensitive to apoptosis while those expressing Hsp27 were resistant to apoptosis induced by Bortezomib (PS-341), a proteasome inhibitor [39]. Silencing of Hsp27 in the resistant lymphoma cells then rendered them susceptible to Bortezomib-induced death, demonstrating its link in resistance to this chemotherapeutic treatment [39]. Thus, a larger patient sample size may reveal if expression of HSP27 is induced by certain chemotherapeutics or in particular subsets of patients and whether there is any correlation to refractory disease, as resistance to chemotherapy is one of the major hurdles in treating CLL [2].

**Figure 7:**
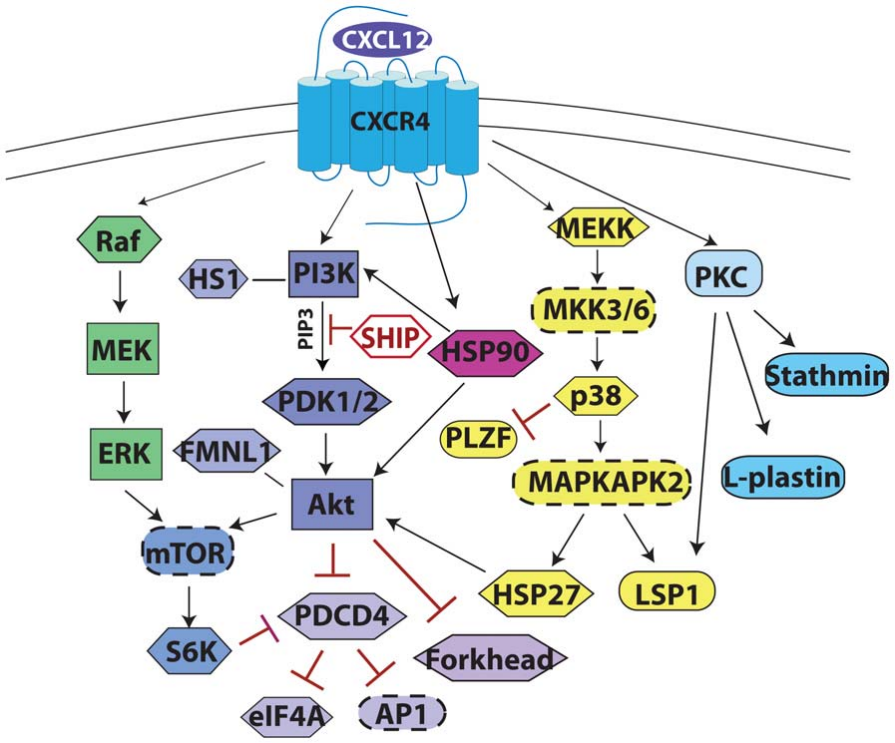
Summary of CXCL12-mediated Signaling in CLL. Signaling diagram depicting pathways activated downstream of CXLC12. Through direct or indirect mechanisms, arrows indicate factors that are activated, red lines ending with a bar indicate factors that are inhibited by the upstream factor, and lines (no arrowhead) indicate interactions. Proteins in hexagons were identified and validated herein or were previously known targets also detected in the LC-MS/MS. Proteins in rectangles are known key signaling molecules of these pathways that were not detected in this LC-MS/MS data set. Proteins in ovals with dashed lines are likely intermediates/targets of the pathways based on previous studies. Proteins in oval shape were also identified by LC-MS/MS but have yet to be validated. Much of our focus has been on the PI3K/Akt and Raf/MEK/ERK pathways due to known implications in CLL cell survival and resistance to apoptosis. Furthermore, the potential involvement of the p38-MAPK pathway in some CLL patients with activation of HSP27 and LSP1 is outlined.

Herein we present follow-up data to PDCD4 and HSP27, although there are numerous other candidate phosphoprotein targets of CXCL12 signaling in CLL cells that have been proposed (Table 2). A summary of our findings from phosphoproteomics analysis combined with some previously established pathways of CXCL12 signaling in CLL are summarized in a signaling diagram (Figure 7). Overall, our data suggests that CXCL12 may preferentially activate survival signaling pathways rather than those involved in cell migration in CLL cells, although some of the pathway components (Gi, Erk, Akt) are common nodes. We have demonstrated that the use of phosphoproteomics is a feasible and informative means of evaluating signaling responses to CXCL12 in CLL, which could be employed for investigating a variety of other stimuli in these or other primary cells. Through phosphoproteomics detection and western blot validation, PDCD4 was found to be a common phosphorylation target of CXCL12-signaling in CLL while HSP27 was present in only a subset of CLL patients. Although our focus was on CXCL12 as a survival factor, it is likely that other growth and survival stimuli may synergistically activate these pathways and downstream targets. Therefore, PDCD4 and HSP27, which have previous implications in regulation of apoptosis and carcinogenesis, may represent potential therapeutic targets for treatment of CLL. In fact, small molecule stabilizers of PDCD4, that enhance its function as a tumor suppressor by inhibiting its degradation, are currently being developed due to its potential as a therapeutic target for numerous cancers. Such agents could prove to be useful agents in combination with other therapeutic modalities for the treatment of CLL [40].

## Supporting Information

Table S1 Table of all phosphoproteins and corresponding phosphopeptides identified by LC-MS/MS analysis on IMAC enriched CLL samples.
(0.18 MB XLS)

Figure S1 Mass spectra of PDCD4 phosphopeptides. Mass spectra from the 3 phosphopeptides from PDCD4 that were identified in the LC-MS/MS analysis. The top spectrum (A) represents the phosphopeptide with Ser457, which is the phosphosite detected by the antibody used in follow-up western blot analysis. B) Spectrum for Ser94 phosphorylation site. C) Spectrum for Ser76 phosphorylation site.
(0.10 MB DOC)

Figure S2 Mass spectrum of HSP27 phosphopeptide. Mass spectrum from the HSP27 phosphopeptide (Ser 82) that was identified in the LC-MS/MS analysis.
(0.04 MB DOC)

Figure S3 Phosphorylation of PAK2 is present but not induced by CXCL12 in CLL cells. A) Mass spectrum of the phosphopeptide K.YLSpFTPPEK.D (Ser141) of PAK2, which was present in all proteomics runs but had fairly even spectral counts (1–3 spectra) in each CXCL12 stimulation time point). B) Representative western blot of PAK2 phosphorylation (Ser141) over 60 min time course of 30 nM CXCL12 stimulation in 3 different CLL patient's cells reflects no changes in phospho-PAK2 upon stimulation, although total phospho-PAK2 levels were variable between different patients' cells. β-actin served as a loading control.
(0.08 MB DOC)
