# Supplementary material for: Elucidating the CXCL12/CXCR4 Signaling Network in Chronic Lymphocytic Leukemia through Phosphoproteomics Analysis
Source: PLoS One. 2010 Jul 22;5(7):e11716. doi: 10.1371/journal.pone.0011716 (PMC2908618; doi:10.1371/journal.pone.0011716)
Supplement: Figure S1 — Mass spectra of PDCD4 phosphopeptides. Mass spectra from the 3 phosphopeptides from PDCD4 that were identified in the LC-MS/MS analysis. The top spectrum (A) represents the phosphopeptide with Ser457, which is the phosphosite detected by the antibody used in follow-up western blot analysis. B) Spectrum for Ser94 phosphorylation site. C) Spectrum for Ser76 phosphorylation site. (0.10 MB DOC) [file pone.0011716.s002.doc]

**Figure S1. Mass Spectra of PDCD4 Phosphopeptides.**

A Ser457

B Ser94

C Ser76
