# Supplementary material for: Elucidating the CXCL12/CXCR4 Signaling Network in Chronic Lymphocytic Leukemia through Phosphoproteomics Analysis
Source: PLoS One. 2010 Jul 22;5(7):e11716. doi: 10.1371/journal.pone.0011716 (PMC2908618; doi:10.1371/journal.pone.0011716)
Supplement: Figure S2 — Mass spectrum of HSP27 phosphopeptide. Mass spectrum from the HSP27 phosphopeptide (Ser 82) that was identified in the LC-MS/MS analysis. (0.04 MB DOC) [file pone.0011716.s003.doc]

**Figure S2.** **Mass Spectrum of HSP27 Phosphopeptide.**
