# Supplementary material for: Elucidating the CXCL12/CXCR4 Signaling Network in Chronic Lymphocytic Leukemia through Phosphoproteomics Analysis
Source: PLoS One. 2010 Jul 22;5(7):e11716. doi: 10.1371/journal.pone.0011716 (PMC2908618; doi:10.1371/journal.pone.0011716)
Supplement: Figure S3 — Phosphorylation of PAK2 is present but not induced by CXCL12 in CLL cells. A) Mass spectrum of the phosphopeptide K.YLSpFTPPEK.D (Ser141) of PAK2, which was present in all proteomics runs but had fairly even spectral counts (1–3 spectra) in each CXCL12 stimulation time point). B) Representative western blot of PAK2 phosphorylation (Ser141) over 60 min time course of 30 nM CXCL12 stimulation in 3 different CLL patient's cells reflects no changes in phospho-PAK2 upon stimulation, although total phospho-PAK2 levels were variable between different patients' cells. β-actin served as a loading control. (0.08 MB DOC) [file pone.0011716.s004.doc]

**Figure S3. Phosphorylation of PAK2 Is Present but Not Induced by CXCL12 in CLL cells.**

A

B
